# Could Jinfukang alleviate the chemotherapy-related adverse effects in non-small cell lung cancer patients? A protocol for a double-blind, randomized controlled trial

**DOI:** 10.1097/MD.0000000000025002

**Published:** 2021-07-16

**Authors:** Xing Zheng, Wenmin Wang, Gefei Wang, Shenghua Liu

**Affiliations:** The Third Affiliated Hospital of Guangzhou Medical University, Guangzhou , Guangdong Province, China.

**Keywords:** chemotherapy, combination therapy, Jinfukang, non-small cell lung cancer, protocol, randomized controlled trial

## Abstract

**Background:**

Lung cancer is the main cause of cancer-related death in the world, and non-small cell lung cancer (NSCLC) accounts for approximately 85% of all lung cancers. Cisplatin and its derivatives are the first-line chemotherapeutic drugs for patients with advanced lung cancer, but the chemotherapy-related adverse reactions greatly impact the quality of life (QOL) of patients and limit their use. Jinfukang is a commonly used traditional Chinese medicine preparation with anti-tumor effect in China, which has been approved by China Food and Drug Administration against NSCLC. At present, there is a lack of strict randomized controlled trials to study whether Jinfukang could alleviate the chemotherapy-related adverse effects in the treatment of advanced NSCLC. Therefore, we intend to perform a double-blind, placebo controlled, randomized trial to evaluate the effect of Jinfukang in alleviating the chemotherapy-related adverse effects of patients with advanced NSCLC.

**Methods:**

This is a prospective, double-blind, randomized, placebo controlled trial. According to the randomized control principle, 168 patients will be divided into treatment group and control group at 1:1 ratio. The patients in the two groups will be treated continuously for 3 cycles and followed up for 3 years. Outcome indicators include: the incidence of chemotherapy-related adverse effects, the progression-free survival (PFS), total effective rate, and QOL evaluation. We will use SPSS19.0 to analyze the results.

**Conclusions:**

This study will help to evaluate the effect of Jinfukang alleviating chemotherapy-related adverse effects in the treatment of advanced NSCLC.

**Trial registration:**

DOI 10.17605/OSF.IO/YWBSC

## 1 Introduction

Lung cancer is the leading cause of cancer-related death worldwide. Every year, about 1.8 million people are diagnosed with lung cancer and about 1.6 million die of it.^[1]^ Lung cancer includes small cell lung cancer (SCLC) and non-small cell lung cancer (NSCLC). Among them, about 85% are NSCLC patients, and 70% of the patients are found to have advanced cancer or tumor metastasis.^[2,3]^ Despite the continuous development of targeted anti-cancer drugs in recent years, cisplatin and its derivatives are still first-line chemotherapeutic drugs for patients with lung cancer, especially for patients with advanced lung cancer.^[4,5]^ Although chemotherapy has significantly improved overall survival, these drugs show chemotherapy-related adverse reactions such as nephrotoxicity, neurotoxicity and gastrointestinal toxicity.^[6]^ Patients may suffer from positively interrelated and co-occur fatigue, sleep disruption, pain, and depressed mood, which could greatly impact the quality of life (QOL) of patients and limit their use in cancer patients with poor health.

In the treatment of cancer, traditional Chinese medicine plays an indispensable role in improving the quality of life of patients after chemotherapy. It has been proved that traditional Chinese medicine could improve health and enhance immune function in cancer patients after chemotherapy.^[7,8]^ Jinfukang is a commonly used traditional Chinese medicine preparation with anti-tumor effect in China, which has been approved by China Food and Drug Administration as a drug for the treatment of non-small cell lung cancer.^[9]^ Jinfukang is composed of extracts of 12 traditional Chinese medicines, such as Astragalus, Radix Adenophora, Asparagus, Ligustrum lucidum, Aesculus vulgaris, etc. It can inhibit the proliferation of tumor cells and enhance immune function.^[10]^ In recent years, Jinfukang has been widely used in combination with chemotherapy for lung cancer, has shown synergistic anti-tumor effect in these tests, which can improve immune function and reduce adverse events in the process of chemotherapy.^[11,12]^

Although Jinfukang has showed positive effect in the treatment of lung cancer in clinic, there are still few strict randomized controlled trials to study whether Jinfukang could alleviate the chemotherapy-related adverse effects in the treatment of advanced NSCLC to improve the QOL of patients. Therefore, we intended to perform a double-blind, placebo controlled, randomized trial to evaluate the effect of Jinfukang in alleviating the chemotherapy-related adverse effects of patients with advanced NSCLC.

## 2 Materials and methods

### 2.1 Study design

**Figure 1:**
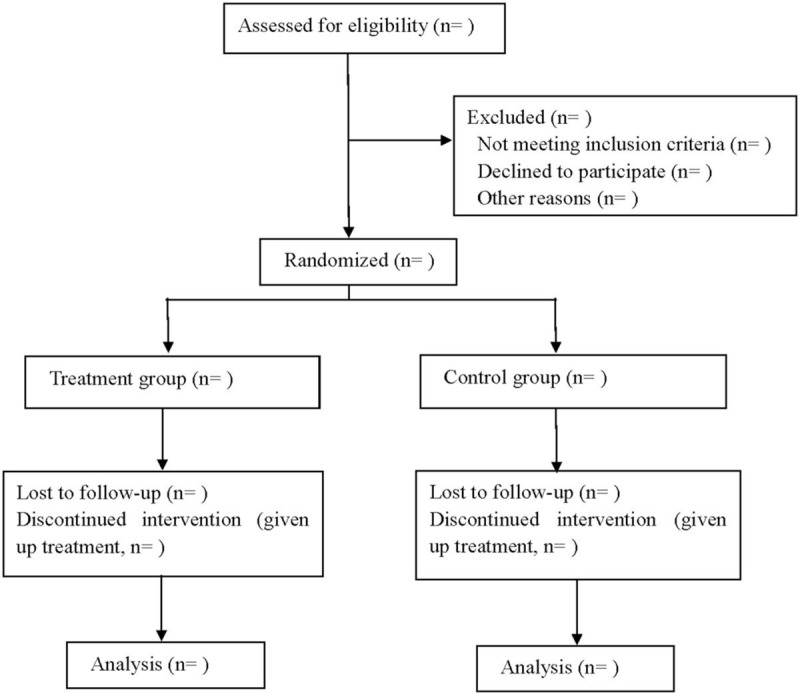
Flow diagram of the study.

This is a prospective, double-blind, randomized, placebo controlled trial. This study program is in line with the Helsinki Declaration and will follow the comprehensive trial reporting standards.^[13]^ The flow chart is shown in Figure 1.

### 2.2 Ethics and registration

This study has been approved by the Clinical Research Ethics Committee of Third Affiliated Hospital of Guangzhou Medical University and registered in open Science Framework (OSF) (Registration number: DOI 10.17605/OSF.IO/YWBSC). Before randomly grouping, all patients are required to sign a written informed consent form. Participants can withdraw from the study at any time for any reason, and whether or not the patient's data are used will be approved by the patient.

### 2.3 Patients

Patients will be included if they meet the following inclusion criteria:

1. diagnosed as stage III b or IV NSCLC by pathological biopsy or immunohistochemistry, older than 18 years old;
2. no contraindications to chemotherapy and willing to accept chemotherapy regimen;
3. Karnofsky Performance Status Scale (KPS) > 60 points;
4. estimated survival time > 6 months;
5. signed informed consent.

Patients will be excluded with the exclusion criteria:

1. with complications such as severe pulmonary infection and need to take other treatment regimens;
2. with severe diseases of liver, kidney, heart, blood system, nervous system and immune system;
3. patients with poor tolerance and compliance;
4. with severe mental illness;
5. with a history of other tumors within 5 years;
6. with allergies or contraindications to medication in this study.

### 2.4 Randomization and blinding

An independent statistician will perform the randomization. Random sequences are generated by using SAS V.9.4 software, and the numbers are stored in opaque envelopes. One hundred patients will be randomly divided into treatment group (Jinfukang group) and control group (Jinfukang-simulation group) according to the rate of 1:1. We will set up special drug managers to be responsible for drug distribution and management, and the allocation of research by attending physicians, patients, assistant researchers, data statistical analysts and nurses is unknown throughout the study.

### 2.5 Intervention

After admission, all patients will be given routine examinations such as blood routine, liver and kidney function, electrolyte and electrocardiogram, and routine treatment such as prophylactic liver protection, gastric mucosal protection and antiemetic, and the intervention contents will be recorded in detail in the pre-designed table. Patients in both groups will receive the same chemotherapy regimen: pemetrexed combined with cisplatin regimen chemotherapy and intravenous infusion of pemetrexed 500 mg/m^2^ and cisplatin 75 mg/m^2^, once a day, 28 days as a cycle.

In the treatment group, at the beginning of the chemotherapy, patients will take Jinfukang oral liquid orally, 30 ml/time, 3 times a day, 28 days as a cycle. While in the control group, patients will receive Jinfukang oral liquid-simulation (the same taste and appearance as the Jinfukang oral liquid).

All patients will receive 3 cycles of treatment, blood routine examination every week, liver and kidney function and ECG every 2 weeks for health assessment. Each treatment cycle is evaluated according to the efficacy evaluation criteria.

### 2.6 Outcome measures

The primary outcomes of this trial are the incidence of chemotherapy-related adverse effects and the progression-free survival (PFS). The secondary outcomes include total effective rate^[14]^ and quality of life evaluation by Karnofsky Performance Status Scale (KPS) and EORTC QLQ-C30 evaluation scale.^[15]^

### 2.7 Data collection and management

The data collection for this study will be specially carried out by two assistant researchers and recorded in a pre-designed form. Two research assistants are followed up. After the treatment is completed, the patients will be followed up once a month in the first year and every 3 months in the second to third years. Progression free survival and overall survival will be recorded. Personal information about potential participants and registered participants will be collected, shared and stored in a separate storeroom to protect the confidentiality before, during and after. Access to the database is limited to the researchers of this research group.

### 2.8 Sample size

According to our preliminary study, the expected incidence of chemotherapy-related adverse effects in treatment group and control group should be 15% and 35, respectively. Assuming a type I error of 5% at 80% power, and a potential dropout of 20%, a total of 168 patients will be recruited.

### 2.9 Statistical analysis

The data will be processed by SPSS19.0 statistical software, and the counting data will be expressed as rate (%). The comparison between groups will be expressed by the test, and the measurement data will be expressed by mean ± standard deviation. The *t*-test will be used for comparison between groups, and Kaplan–Meier analysis will be used to analyze the survival curve. And *P* *<* .05 is considered statistically significant.

## 3 Discussion

Lung cancer has now become the first incidence of malignant tumor in men and the second in women in the world, and the mortality rate is the first among all malignant tumors, and shows a rising trend.^[16]^ Due to uncontrollable cell proliferation or tumor metastasis, it is estimated that only 16.8% of lung cancer patients survived for 5 years after the first diagnosis.^[17]^ Although the survival time of patients has been prolonged with the progress of treatment methods such as surgery, chemotherapy and radiotherapy, drug side effects, pain and drug resistance are still major problems faced by tumor patients and doctors.^[18,19]^

Traditional Chinese medicine has been widely used in cancer treatment in China. It can not only directly target tumor cells, but also improve the health status of patients to support chemotherapy or radiotherapy.^[20]^ Jinfukang is a traditional Chinese medicine formula, which has been used in the treatment of lung cancer patients for more than ten years, and has achieved good results.^[11,21]^ Modern studies have found that Jinfukang has the effects of inhibiting tumor growth, anti-tumor metastasis and regulating cellular immunity. Although there are obvious side effects and drug resistance, cisplatin is still an important drug in first-line chemotherapy for NSCLC. In recent years, combined with a new drug pemetrexed to improve the efficacy and reduce side effects. But the results are not satisfactory.^[22]^ In vitro studies have found that the combination of Jinfukang and cisplatin can regulate genes related to apoptosis-related signaling pathways and synergistically induce apoptosis of lung cancer cells.^[23]^ A lot of evidences have shown the advantages of Jinfukang in the treatment of lung cancer, but there is a lack of strict clinical research to study whether it could alleviate the chemotherapy-related adverse effects. Therefore, this randomized controlled trial attempts to evaluate the effect of Jinfukang in alleviating the chemotherapy-related adverse effects of patients with advanced NSCLC.

## Author contributions

**Data curation:** Xing Zheng, Wenmin Wang.

**Funding acquisition:** Shenghua Liu.

**Investigation:** Gefei Wang.

**Resources:** Gefei Wang.

**Software:** Wenmin Wang.

**Supervision:** Shenghua Liu.

**Writing – original draft:** Xing Zheng, Wenmin Wang.

**Writing – review & editing:** Xing Zheng, Shenghua Liu.

## References

[1] Zhou X Li Y Zhang M Hao J Gu Q Liu H. Spectrum-effect relationship between UPLC fingerprints and antilung cancer effect of Si Jun Zi Tang. Evid Based Complement Alternat Med 2019;2019:7282681. 31662780 10.1155/2019/7282681 PMC6778903

[2] Wang JJ Wang YL Ge XX Xu MD Chen K Wu MY. Prognostic values of platelet-associated indicators in resectable lung cancers. Technol **Cancer** Res Treat 2019;18:1533033819837261. 30871415 10.1177/1533033819837261 PMC6421614

[3] Wang L Wu W Zhu X Ng W Gong C Yao C. The ancient Chinese decoction Yu-Ping-Feng suppresses orthotopic Lewis lung cancer tumor growth through increasing M1 macrophage polarization and CD4(+) T cell cytotoxicity. Front Pharmacol 2019;10:1333. 31780946 10.3389/fphar.2019.01333 PMC6857089

[4] Ramalingam S Belani C. Systemic chemotherapy for advanced non-small cell lung cancer: recent advances and future directions. Oncologist 2008;13:05–13. 10.1634/theoncologist.13-S1-5 18263769

[5] Dempke WC. Targeted therapy for NSCLC—a double-edged sword? Anticancer Res 2015;35:2503–12. 25964523

[6] Wei D Wang L Chen Y Yin G Jiang M Liu R. Yangyin Fuzheng decoction enhances anti-tumor efficacy of cisplatin on lung cancer. J Cancer 2018;9:1568–74. 29760794 10.7150/jca.24525 PMC5950585

[7] Zhu X Zhou Y Xu Q Wu J. Traditional Chinese medicine Jianpi Bushen therapy suppresses the onset of pre-metastatic niche in a murine model of spontaneous lung metastasis. Biomed Pharmacother 2017;86:434–40. 28012398 10.1016/j.biopha.2016.12.013

[8] Li J Wang JC Ma B Gao W Chen P Sun R. Shenqi Fuzheng injection for advanced gastric cancer: a systematic review of randomized controlled trials. Chinese J Integ Med 2015;21:71–9. 10.1007/s11655-014-1768-8 25246138

[9] Lu J Chen J Kang Y Wu J Shi H Fu Y. Jinfukang induces cellular apoptosis through activation of Fas and DR4 in A549 cells. Oncology Lett 2018;16:4343–52. 10.3892/ol.2018.9149 PMC6126349 30197670

[10] Zujun Que Bin Luo Fangfang Qian. Jinfukang induced senescence of circulating tumor cells in lung cancer by p16/RB signal pathway. Tumor 2018;38:215–21.

[11] Zengliang Li Banqing Pan Xiaoyue Wang. Clinical study on Jinfukang oral liquid combined with pemetrexed in treatment of non-small cell lung cancer. Drugs & Clinic 2019;34:1827–30.

[12] Xiaoming Huang Jian Sun Aihua Hou. Effect and mechanism of Jinfukang oral liquid in increasing sensitivity of human lung adenocarcinoma PC9/R cells to gefitinib. Drug Eval Res 2020;43:676–82.

[13] Schulz KF Altman DG Moher D. CONSORT 2010 statement: updated guidelines for reporting parallel group randomised trials. BMJ 2010;340:c332. 20332509 10.1136/bmj.c332 PMC2844940

[14] WHO handbook for reporting results of cancer treatment. Geneva, Switzerland: World Health Organization Offset Publication; 1979.

[15] Fayers P Bottomley A. Quality of life research within the EORTC-the EORTC QLQ-C30. European Organisation for Research and Treatment of Cancer. Eur J Cancer 2002;38:S125–33. 11858978 10.1016/s0959-8049(01)00448-8

[16] Li C Hong W. Research status and funding trends of lung cancer biomarkers. J Thoracic Dis 2013;5:698–705. 10.3978/j.issn.2072-1439.2013.10.10 PMC3815715 24255784

[17] DeSantis C Ma J Bryan L Jemal A. Breast cancer statistics, 2013. CA: Cancer J Clin 2014;64:52–62. 24114568 10.3322/caac.21203

[18] Shanker M Willcutts D Roth JA Ramesh R. Drug resistance in lung cancer. Lung Cancer (Auckl) 2010;1:23–36. 28210104 PMC5312467

[19] Cassileth BR Rizvi N Deng G Yeung KS Vickers A Guillen S. Safety and pharmacokinetic trial of docetaxel plus an Astragalus-based herbal formula for non-small cell lung cancer patients. Cancer Chemother Pharmacol 2009;65:67–71. 19421753 10.1007/s00280-009-1003-z PMC3746541

[20] Cao C Han D Su Y Ge Y Chen H Xu A. Ginkgo biloba exocarp extracts induces autophagy in Lewis lung cancer cells involving AMPK /mTOR /p70S6k signaling pathway. Biomed Pharmacother 2017;93:1128–35. 28738521 10.1016/j.biopha.2017.07.036

[21] Qiming W Chong L Zhiwei C Xuming Y. Network-based analysis of Jinfukang in the treatment of lung cancer. J Eur J Integ Med 2020;(prepublish).

[22] Qin X Yu S Zhou L Shi M Hu Y Xu X. Cisplatin-resistant lung cancer cell-derived exosomes increase cisplatin resistance of recipient cells in exosomal miR-100-5p-dependent manner. Int J Nanomed 2017;12:3721–33. 10.2147/IJN.S131516 PMC5439933 28553110

[23] Lu J Chen J Xu N Wu J Kang Y Shen T. Activation of AIFM2 enhances apoptosis of human lung cancer cells undergoing toxicological stress. Toxicol Lett 2016;258:227–36. 27392435 10.1016/j.toxlet.2016.07.002

